# Cultural Adaptation and Selection of a Minimal Set of Variables from Two Adolescent Pregnancy Risk Instruments (IRENE and REND) in Colombian Schoolgirls

**DOI:** 10.3390/nursrep16070248

**Published:** 2026-07-16

**Authors:** Nancy Milena Sepúlveda-Sepúlveda, Carolina Vargas-Porras, María Inmaculada De Molina-Fernández, Zayne Milena Roa-Díaz

**Affiliations:** 1 Nursing Program, Universidad Cooperativa de Colombia, Bucaramanga 680006, Colombia; 2 Department of Nursing, Rovira i Virgili University, 43002 Tarragona, Spain; 3 Faculty of Health, School of Nursing, Universidad Industrial de Santander, Bucaramanga 680002, Colombia; carvarpo@uis.edu.co; 4 Instituto Proinapsa, Universidad Industrial de Santander, Bucaramanga 680006, Colombia; zayne.roa@correo.uis.edu.co

**Keywords:** adolescent pregnancy, pregnancy risk screening, instrument validation, cultural adaptation, content validity, factor analysis, dimensionality reduction, nursing, adolescents, Colombia

## Abstract

**Background/Objectives**: Adolescent pregnancy remains a global public health challenge associated with adverse outcomes for both mothers and newborns. This study aimed to use two instruments and several multivariate techniques to identify a minimal set of variables that reproduces the instrument-based classification of adolescent pregnancy risk, while remaining parsimonious with the original instruments. **Methods**: A cross-sectional, quantitative methodological study was conducted among Colombian schoolgirls, comprising 160 adolescents in the face-validity phase and 319 in the risk-estimation and modeling phase. The IRENE and REND instruments, originally developed to assess the risk of adolescent pregnancy, underwent cultural adaptation, face validity and content validity. Subsequently, the instruments were administered to estimate the risk. Finally, Factor Analysis and Categorical Principal Component Analysis were applied as exploratory dimensionality reduction techniques to identify the most relevant variables for retention, thereby preserving the parsimony of the original versions. **Results**: Overall, 80.3% of participants were classified as not at risk, while the remainder were classified as at risk by one or both instruments. The results suggest that variables such as who the adolescent lives with, the age at which the adolescent had their first complete sexual intercourse, and how the adolescent would respond to an unplanned pregnancy are factors associated with the instrument-based risk classification. Subsequently, dimensionality reduction and logistic regression analyses identified a small subset of variables that can be used to reproduce the instrument-based classification of adolescent pregnancy risk. **Conclusions**: The reduced set of variables reproduced the instrument-based risk classification with high internal accuracy. Because the predictors and the outcome derive from the same instruments, these findings reflect internal reproduction of the instrument classification rather than prediction of an observed pregnancy, and they require external validation. Overall, the reduced and optimized set of variables from the IRENE and REND instruments offers a parsimonious approach that reproduces the instrument-based risk classification and that may support rapid screening once validated in independent adolescent samples. The main limitations are the use of a single institution, the non-probabilistic convenience sample, and the absence of an observed pregnancy outcome.

## 1. Introduction

Adolescent pregnancy is defined as an early, unplanned, or unintended pregnancy [1]. In terms of age, adolescence is conventionally defined as the second decade of life, from 10 to 19 years [2]; adolescent pregnancy therefore refers to a pregnancy occurring within this age range, and the present study focused on older adolescents aged 15 to 19 years. Although epidemiological data indicate that the adolescent fertility rate, expressed as births per 1000 women aged 15 to 19 years, decreased from 64.5 to 41.3 between 2000 and 2023 [3], adolescent pregnancy remains a global public health problem [4,5,6].

Each year, approximately 21 million adolescents experience an unplanned pregnancy, and 12 million become mothers, especially in low- and middle-income countries where educational opportunities and access to information and services remain limited [3,7]. Adolescent pregnancy is a major challenge because of its health, social, and economic consequences [8]. At the maternal level, adolescent pregnancy is associated with severe health risks due to biological immaturity. These include malnutrition, anemia, urinary infections, hypertension disorders, hemorrhage, premature rupture of membranes, preterm birth, and maternal mortality. For the children, these complications include low birth weight and infant mortality [3,9]. It also contributes to school dropout, reduced academic and personal opportunities, and the perpetuation of poverty across generations [3,6].

Evidence-based strategies to prevent adolescent pregnancy include comprehensive sexuality education and improved access to effective contraception, in line with World Health Organization guidance for preventing early pregnancy [10] and with current medical eligibility criteria for contraceptive use [11]. Early identification of adolescents at higher risk can support the timely delivery of these preventive interventions. Previous studies have evaluated these risk factors, consistently identifying early sexual initiation, multiple partners, inconsistent contraceptive use, low parental education, and family structure as determinants of adolescent pregnancy [12,13]. Identifying risk factors for adolescent pregnancy and developing instruments that support early risk classification remain major challenges for public health and clinical practice because doing so may facilitate timely intervention. Several instruments have been designed to assess pregnancy intention and its related constructs. For example, in the United States, there exist the National Survey of Family Growth (NSFG) and the Demographic and Health Surveys (DHS); the latter classifies pregnancies into categories such as intended, mistimed, unwanted, and unintended [14,15]. The London Measure of Unplanned Pregnancy (LMUP) is a validated instrument designed primarily for the retrospective assessment of pregnancy planning or intention in current or recent pregnancies [16]. Other instruments, such as the Risk of First-Time Adolescent Pregnancy (RAP) [17], the Timing-based Measure of Unintended Pregnancy (TMUP) [18], the Unintended Pregnancy Risk Index (UPRI), and the Desire to Avoid Pregnancy (DAP) scale [19], were devised in the last decade; however, they largely lack extensive validation within adolescent populations and across diverse socio-cultural contexts.

It is important, moreover, to distinguish related but non-equivalent constructs that these instruments address. Whereas pregnancy intention and unintended or mistimed pregnancy refer to the cognitive and affective orientation toward a pregnancy, frequently assessed retrospectively [20], unplanned pregnancy captures the degree of planning that precedes conception [21], and the desire to avoid pregnancy reflects a prospective preference rather than an observed outcome [19]. In contrast, the present study addresses the prospective risk classification produced by IRENE and REND, which should not be equated with the actual occurrence of a future pregnancy. Consequently, IRENE and REND are treated here as risk classification instruments, and their output is interpreted accordingly throughout this work.

Some instruments have more recently been evaluated in new contexts; for example, the DAP scale showed favorable psychometric performance and validity in the United Kingdom [22]. In Spain, two instruments were designed to assess the risk of adolescent pregnancy: the Instrumento de valoración del Riesgo de Embarazo No Esperado (IRENE—Unexpected Pregnancy Risk Assessment Instrument), specific to women without prior experience of pregnancy [23], and the Riesgo de Embarazo no Deseado (REND—Unwanted Pregnancy Risk) instrument for women aged 15 to 45 years, with or without prior experience of pregnancy [24].

Despite invaluable advances in this area, instruments specifically adapted or validated to measure adolescent pregnancy risk in Latin America remain limited, which underlines the relevance of the present study. Latin America and the Caribbean have one of the highest adolescent fertility rates in the world and remain second only to Sub-Saharan Africa [3,25]. Some estimates indicate that the regional rate is 48% higher than the global average [26]. In this regard, the use of instruments to assess and classify the risk of adolescent pregnancy in Latin America is a current issue of high relevance.

Although IRENE and REND were developed in Spain, they provide a reasonable starting point for the Colombian setting for three reasons: both were designed specifically to classify the risk of adolescent pregnancy rather than general pregnancy intention; both operationalize determinants repeatedly reported in adolescent populations, such as early sexual initiation, contraceptive use, low parental education, and family structure [12,13]; and both are written in Spanish, which reduces the linguistic distance for adaptation. Nevertheless, cultural and linguistic proximity cannot be assumed from a shared language alone, so it is treated here as a hypothesis to be examined rather than a premise, which is precisely why both instruments underwent cultural adaptation and content validation before being administered. However, as with many tools, they depend on a considerable number of variables, which can make them confusing and limit their use, particularly where more concise practical tools are necessary [27]. This could provide an additional pathway to strengthen nursing practice in adolescent sexual and reproductive health through timely and effective preventive interventions [28]. The specific gap addressed in this study is therefore the absence of brief, culturally adapted instruments suitable for the rapid classification of adolescent pregnancy risk in this context, rather than their full psychometric validation or the prediction of observed pregnancy. In this regard, this study, conducted in Colombia and based on the REND and IRENE instruments, aimed to use various multivariate techniques to identify a minimal set of variables that could reproduce the instrument-based classification of adolescent pregnancy risk, while remaining parsimonious with the original instruments.

## 2. Materials and Methods

### 2.1. Study Design, Setting, and Participants

This was a cross-sectional, methodological study involving the cultural adaptation, face and content validity, and item reduction in two adolescent pregnancy risk instruments, with data collected at a single time point for the adaptation, risk classification, and modeling; a limited 12-month follow-up was subsequently attempted for descriptive purposes only and was not used for model development or validation. The primary outcome was the instrument-defined adolescent pregnancy risk classification (at risk versus not at risk), as determined by IRENE and REND. The methodological outputs were the culturally adapted instruments, their face and content validity, and the minimal set of variables retained for each instrument together with the classification performance of the corresponding logistic models. This study was conducted in Bucaramanga, a medium-sized city in northeastern Colombia. In 2023, adolescent births accounted for 11.6% of all registered births in the city, including births to mothers aged 10 to 14 years and 15 to 19 years (own calculations based on DANE, Vital Statistics on Births and Deaths, 2024). This percentage is slightly higher than in cities such as Bogotá (8.7%), Cali (10.2%) and Medellín (10.4%) (own calculations based on DANE, Vital Statistics on Births and Deaths, 2024).

**Figure 1:**
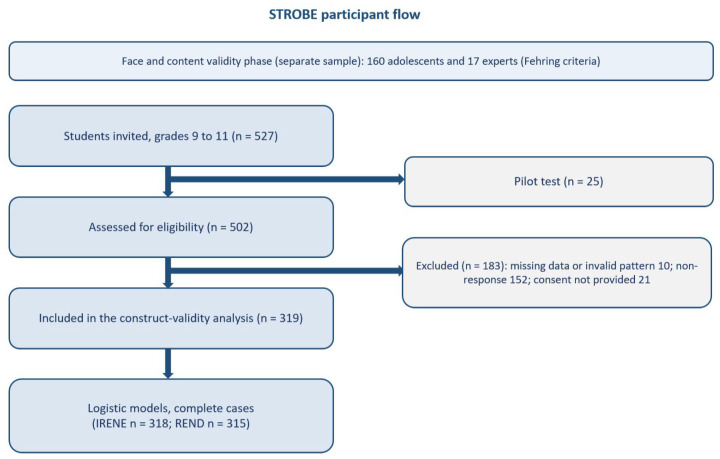
Flow diagram of the study. Of 527 female high-school students invited (grades 9 to 11), 25 took part in the pilot test; of the 502 assessed for eligibility, 183 were not included (missing data or invalid response patterns, 10; non-response, 152; consent not provided, 21), leaving 319 adolescents in the risk-estimation and modeling analyses. The face- and content-validity phase involved a separate sample of 160 adolescents and 17 experts (Fehring criteria). Complete-case binary logistic models were fitted for IRENE (*n* = 318) and REND (*n* = 315), with 10-fold cross-validation and Firth penalized estimation.

The study was conducted in one of the largest educational institutions in Bucaramanga, which is characterized by serving a student population from highly diverse socioeconomic and social backgrounds. Eligible participants were female high-school students aged 15 to 19 years enrolled at the institution during data collection. Adolescents with verbal or written communication difficulties that could compromise self-report validity were excluded, as was one adolescent who was pregnant at the time of data collection; a previous history of pregnancy was not an exclusion criterion. Data were collected in 2023 using non-probabilistic convenience sampling. Analyses followed a complete-case approach, so that only adolescents with complete responses on the items required for a given analysis were included in that analysis; the number of participants retained at each phase is reported in the study flow diagram (Figure 1). Of 527 students invited, 25 took part in the pilot test; of the remaining 502 assessed for eligibility, 183 were not included in the analytic sample (10 for missing data or invalid response patterns, 152 for non-response, and 21 because consent was not provided), leaving 319 adolescents for the risk-estimation and modeling analyses, whereas 160 adolescents had participated in the adolescent face-validity phase. Table 1 presents the participants’ sociodemographic characteristics. The mean age of the participants was 15.4 years (SD 0.7), and all were aged between 15 and 18 years; although the inclusion criterion spanned 15 to 19 years in line with the literature, no participant was older than 18.

Recruitment proceeded as follows. Three public educational institutions in Bucaramanga were first contacted by email through their principals; after several meetings, one institution agreed to participate. Eligible grades were identified in a meeting with the academic coordination of the ninth, tenth, and eleventh grades, where adolescents of the target age were expected; each grade comprised between one and eight classrooms of approximately 40 students. The research team then visited each classroom, explained the study, and distributed the parental consent and adolescent assent forms; instruments were collected individually until the required sample size was reached.

### 2.2. Cultural Adaptation

Based on the original IRENE and REND instruments, terms with different meanings in the source population, Spain, and the target population, Colombia, were identified and replaced to improve comprehension. Semantic, conceptual, and content equivalence were assessed, and final adjustments were made by consensus following international guidelines [30]. Three experts participated in this phase; two held a PhD in nursing, and all had experience working with adolescents. This small expert committee is adequate for the semantic and cross-cultural reconciliation step, consistent with international guidelines for test adaptation [30], whereas the formal quantification of content validity relied on the larger panel of 17 experts described below. Because both the source (Spain) and target (Colombia) versions share the Spanish language, forward and back translation were not applicable; the procedure therefore consisted of a semantic and cross-cultural adaptation of terms, with the final wording established by expert consensus, and item comprehension was subsequently evaluated empirically in adolescents during the face-validity phase. No formal cognitive interviews were conducted; however, a structured pilot test with 25 adolescents was carried out, which showed that some REND items lacked an applicable response option for adolescents who had not had sexual intercourse. As a result, item 11 of REND was reworded from coital to complete sexual intercourse and a No, I have never had complete sexual intercourse response option was added. The original and adapted wording of every modified item is provided in Supplementary Table S1.

### 2.3. Face and Content Validity

Face validity was assessed in 160 adolescents who met the same inclusion criteria described above. Data for this phase were collected in September 2022 using non-probabilistic sampling, and sample size was determined according to Anthoine et al. [31] (ten times the number of items: IRENE, 10 × 8 = 80; REND, 10 × 16 = 160; the larger value, 160, was adopted). Item comprehension was evaluated after the linguistic adjustments made during cultural adaptation. In addition, 17 experts were recruited according to Fehring’s criteria [32] through the database of the Colombian Ministry of Science, Technology and Innovation. The experts were university professors and/or professionals affiliated with institutions that promote sexual and reproductive health and were contacted by email. In the assessment of face validity, they assessed item comprehension, clarity and accuracy, including the response options. Accordingly, this stage comprised two complementary components: an adolescent face-validity assessment in 160 schoolgirls, centered on item comprehension, and an appraisal by 17 experts that covered comprehension, clarity, and accuracy for face validity and pertinence and relevance for content validity.

For content validity, they assessed item pertinence and relevance using an ordinal scale. Item-level and global content validity index values were calculated for each instrument. Items with a CVI lower than 0.78 were reviewed, and those with values lower than 0.50 were excluded [33]. Final wording changes were made by expert consensus to improve comprehension, clarity and accuracy [33,34]. Comprehension, clarity and accuracy were each rated on a three-point ordinal scale (1 = understood, 2 = partially understood, 3 = not understood, with the corresponding wording for clarity and accuracy), and the results were classified as high (≥85%), medium (80–84.9%) or low (≤79%). Pertinence and relevance were rated by the experts on a four-point ordinal scale (1 = not, 2 = slightly, 3 = pertinent or relevant, 4 = very pertinent or relevant); the item-level content validity index was computed as the proportion of experts rating an item 3 or 4, and the scale-level index as the average across items, following Polit, Beck and Owen [33]. No item reached the exclusion threshold: all items were retained (eight in IRENE and sixteen in REND), and the changes consisted only of linguistic adaptation and semantic adjustment of the wording. The item-level pertinence and relevance indices are reported in Supplementary Table S2.

It is important to note that the group of participants involved in face and content validity was independent of the sample selected for the pregnancy risk estimation. Consequently, their responses were not included in the risk estimation analysis.

### 2.4. Estimating the Risk of Pregnancy in Adolescents

To estimate the risk of pregnancy in adolescents, two tools were used: IRENE comprised eight polytomous items grouped into five dimensions: (1) parents’ educational level, (2) family APGAR, (3) sexual behavior, (4) contraceptive use and (5) pregnancy desire. Risk is classified when at least three dimensions are affected; when two affected dimensions include sexual behavior, contraceptive use or pregnancy desire; or when the affected dimensions correspond to the combinations (3 and 4), (3 and 5), (4 and 5), (1, 2 and 3), (1, 2 and 4) or (1, 2 and 5) [23]. REND comprises 16 items distributed across three dimensions: (i) sexual behavior and habits, (ii) pregnancy intention, and (iii) contraceptive knowledge and use. Most items are polytomous (*n* = 13), whereas three are dichotomous. Risk is determined by summing item scores to a maximum of 29, with higher scores indicating greater risk [24]. An adolescent was classified as at risk when the REND total score was 12 or higher. A detailed description of the items, dimensions, coding, scoring direction and risk thresholds of both instruments is provided in Supplementary Tables S3 and S4. For adolescents who reported never having had complete sexual intercourse, the items that did not apply were answered through the dedicated response option added during the cultural adaptation, so that every participant could complete the instruments. This non-applicable response option was scored as zero, the lowest-risk value.

### 2.5. Critical Variable Selection

After estimating the risk of adolescent pregnancy using both the IRENE and REND instruments, a set of multivariate statistical analyses were conducted to reduce the number of explanatory variables. Factor Analysis (FA) and Categorical Principal Component Analysis (CATPCA) were used as exploratory dimensionality reduction techniques to identify variables suitable for retention in each instrument [35]. Because several variables were categorical and did not meet the assumptions usually required for conventional factor analytic approaches, CATPCA was included as a complementary strategy. Conventional factor analysis was therefore used only as an exploratory and descriptive heuristic to inspect the dimensional structure; because it assumes continuous and approximately normal variables, its solutions for the polytomous items are interpreted with caution, and approaches based on polychoric correlations, together with CATPCA, are more appropriate for ordinal data [36].

For FA, Bartlett’s test of sphericity and the Kaiser-Meyer-Olkin (KMO) measure were used to assess the suitability of the correlation matrix. The number of retained dimensions was determined using eigenvalues of 1 or greater and explained variance, and the retained dimensions were rotated using the Varimax orthogonal method for interpretative clarity. Orthogonal rotation was chosen for interpretive simplicity; because the final step retained a single variable per dimension rather than factor scores, the choice between orthogonal and oblique rotation has limited influence on the variables selected, and the likely correlation among domains is acknowledged in the interpretation. Communalities were also examined to assess the extent to which items were explained by common factors. The principal components extraction method was used. Given that the CATPCA poses difficulties for identifying the optimal number of components to be retained, several analyses were carried out considering between two and five components for IRENE and REND, with five being proposed in the original version of IRENE, and three in the original version of REND. For both the FA and the CATPCA, total variance explained by the factors was used as a measure of model goodness-of-fit. Because the retained dimensions suggested correlated and potentially overlapping information across items, one variable per dimension was selected to continue the analysis. The selected variables were those with the highest factor loadings in both FA and CATPCA solutions for each instrument. The selected variables were then entered as predictors into two binary logistic regression models, one for IRENE and one for REND, to reduce redundancy and mitigate multicollinearity. The selection of the highest-loading item per dimension follows the rationale that the most strongly loading indicator is the most representative marker of its factor, a criterion used when shortening scales [37]; nevertheless, because reliance on statistical loadings alone can narrow content coverage in short forms [38], this reduction is regarded as exploratory and parsimony-oriented, and the content representativeness of the retained items, beyond the expert content-validity appraisal already performed, should be confirmed before applied use.

In order to address the optimism that arises from fitting and evaluating the models in the same sample, the discrimination of each logistic model was assessed through 10-fold cross-validation, and calibration was examined with the Hosmer–Lemeshow test. In addition, given the small number of at-risk cases and the quasi-complete separation observed for some categories, the models were re-estimated using Firth penalized logistic regression [39,40], and the associations are reported as odds ratios with 95% confidence intervals. Combining sparse categories was considered as an alternative, but penalization was preferred in order to preserve the original response categories of the instruments. Analyses were conducted on complete cases; the instrument-based risk classification was available for all 319 adolescents for IRENE, with 318 contributing complete predictor data to the model, and for 315 adolescents for REND, so that the models and confusion matrices are based on these analytic samples.

The dependent variable (*Gi*) in both models was the previously calculated instrument-defined pregnancy risk. It is important to note, in this respect, that this instrument-defined outcome does not correspond to a clinically verified or prospectively observed pregnancy, so that it is interpreted as a classification rather than as a prediction of future pregnancy. The dependent variable is coded as follows, where

*Gi* = 0, not at risk of pregnancy (*G1*).

*Gi* = 1, at risk of pregnancy (*G2*).

Considering that

$P\left(Gi=1\right)\;=\;\frac{1}{1+exp\left(-z\right)}$, then

$z\;=\;\beta_{0}\;+\;\beta_{1}X_{1}\;+\;\beta_{2}X_{2}\;+\;\ldots \;+\;\beta_{k}X_{k}$, where

*P* is the probability of belonging to *Gi* = 1 with respect to *Gi* = 0, and X_1_, …, X_k_ are predictors selected from FA and CATPCA. Each of the two logistic models used, as its dependent variable, the risk classification produced by its corresponding instrument (IRENE or REND); the combined grouping defined next was used only to summarize overall risk across both instruments. Adolescents classified as not at risk by both instruments (IRENE and REND) were assigned to *G1*, whereas those classified as at risk by either instrument or by both were assigned to *G2*. Model’s performance was evaluated using Nagelkerke’s R^2^ and the classification table (confusion matrix). Statistical analyses were performed in STATA 15 and Orange Data Mining 3.39.0.

### 2.6. Ethical Considerations

The study was approved by the Bioethics Committee of the Universidad Cooperativa de Colombia (BIO173). All participants were informed about study objectives and procedures. For participants younger than 18 years of age, written informed consent was obtained from a parent or legal guardian, along with the participant’s assent. Participants aged 18 years or older provided written informed consent directly. Participation was voluntary and anonymous. To reduce any sense of coercion, participation carried no academic incentive or penalty; adolescents could decline to take part or withdraw at any time without any consequence for their schooling, and teachers were not involved in administering the instruments or in handling the responses. Given the sensitive nature of the questions on sexual and reproductive health, the instruments were completed privately and anonymously, and participants were free to omit any item they did not wish to answer. The questionnaires were self-administered, and the responses carried no personal identifiers. Adolescents completed the instruments in approximately 15 min, generally in recreational areas outside the classroom to protect their privacy, although those who preferred to remain in the classroom were allowed to do so; doubts were clarified, and all data were collected by the principal investigator. A referral pathway was established whereby any adolescent identified as vulnerable, or who requested emotional support because of the sensitivity of the items, would be referred immediately to the school counselling service (psicorientación); no referral proved necessary during the study.

## 3. Results

### 3.1. Cultural Adaptation

Based on cultural adaptation, three items of the IRENE instrument were modified: items 4, 5, and 7. For the REND instrument, nine items (1, 2, 3, 4, 11, 12, 13, 14 and 16), were modified because some expressions were uncommon in the Colombian context. For example, in items 1, 2, 3, 4, and 11, terms referring to “coital” were replaced with “complete” to improve comprehension and linguistic appropriateness; in this study, “complete” refers to penetrative sexual intercourse. This wording was adopted because, during the pilot and the face-validity assessment, the original term “coital” was poorly understood by the adolescents, whereas “relación sexual completa” is the locally familiar expression for penetrative vaginal intercourse; we acknowledge that this colloquial expression carries a coitocentric connotation and use it only to maximize comprehension in the target population, without implying that non-penetrative practices are less valid. The exact original and adapted wording of every modified item is reported in Supplementary Table S1. In addition, the term “varón” (male) was replaced with “hombre” (man) to align with local colloquial usage.

### 3.2. Face Validity and Content Validity

In expert face validity, IRENE showed low comprehension (76%), low clarity (71%) and low accuracy (71%), whereas REND showed high comprehension (89%), high clarity (85%) and average accuracy (83%). In the adolescent group, face validity showed a high level of comprehension for both instruments (IRENE 91% and REND 94%). The low expert ratings of comprehension, clarity, and accuracy for IRENE referred to its original wording and were precisely what motivated the linguistic adjustments made during the cultural adaptation; after those adjustments, the adolescents showed high comprehension of both instruments. Pertinence and relevance, in turn, concern whether the items adequately represent the construct of adolescent pregnancy risk, a property distinct from the clarity of the initial wording, which is why both instruments were judged favorable on content validity despite the initial observations on IRENE. In content validity, experts rated both instruments as pertinent (S-CVI: IRENE 0.98, REND 0.82) and relevant (S-CVI: IRENE 0.99, REND 0.83).

### 3.3. Pregnancy Risk Assessment

Through IRENE and REND, it was established that 80.3% of female adolescents are not at risk of pregnancy (group G1). The remaining participants were classified as at risk by one or both instruments (G2). Both instruments classified 10.8% of adolescents as at risk. However, 2.2% were classified as at risk by IRENE but not by REND, whereas 6.7% were classified as at risk by REND but not by IRENE (Table 2). The cross-tabulation that follows summarizes this concordance and discordance; REND classified a larger proportion of adolescents as at risk than IRENE. The instruments do not increase or reduce risk; rather, they classify participants differently.

### 3.4. Critical Variable Selection

#### Variables Reduction

Regarding the suitability of the data for FA, the KMO was 0.64 for IRENE and 0.86 for REND. The KMO for IRENE (0.64) is modest, indicating only mediocre sampling adequacy, so its factor solution is interpreted with caution, whereas the value for REND (0.86) is meritorious; the negative loadings observed for some items, such as condom use, reflect inverse associations with the underlying factor rather than a problem of fit. Bartlett’s test of sphericity was significant for both instruments (*p* < 0.001). Table 3 presents the IRENE factor loading matrices obtained with FA and CATPCA. In IRENE, CATPCA accounted for 71.3% of the total explained variance, whereas FA accounted for 57.1% across three dimensions. In both analyses, the item “Are you currently trying to get pregnant?” presented low factor loading.

In both analyses, the IRENE items were organized into three factors. In the first factor, item 6 “With how many different people have you had complete sexual intercourse?”, item 5, “At what age did you have your first complete sexual intercourse?”, and item 7 “Do you use a condom for complete sexual intercourse?” showed high loadings, although the latter loaded negatively. The second factor grouped the items related to parents’ educational level (items 1 and 2). The third factor grouped item 4, “What are your family relationships like?”, positively, whereas item 3, “Do you live with your parents?”, loaded negatively. Based on the highest factor loadings in both FA and CATPCA, one variable was selected from each factor:

From factor 1: IRENE i5; From factor 2: IRENE i1; From factor 3: IRENE i3.

Table 4 presents the REND factor loading matrices obtained with FA and CATPCA. In REND, five factors were retained, accounting for 67.1% of the total explained variance in FA and 69.2% in CATPCA. Across both analyses, the items were not grouped uniformly or in exactly the same factors as in the original version. In both analyses, items 1, 3, 4, 5 and 6 loaded onto factor 1, although item 6 did so negatively. Item 7, “If you got pregnant without wishing to, what decision would you make?”, loaded onto factor 5. In contrast, the items “In your current situation, would you like to become pregnant?” and “Do you think that, in your current situation, it would be the right time to become pregnant?” loaded onto factor 3 in FA and factor 2 in CATPCA. Item 2, “Did you use any contraceptive method during your first complete sexual intercourse?” loaded onto different factors in the two analyses. These departures from the original three-dimensional structure are interpreted with caution, since they may reflect the younger and more homogeneous adolescent sample and the ordinal nature of the items rather than a stable alternative structure; accordingly, the dimensional reduction is treated as exploratory and its implications for construct validity are taken up in the Discussion.

Item 10, “If you have been pregnant before, was the pregnancy planned?”, showed a low factor loading because all adolescents responded negatively. Because all adolescents answered negatively, no participant in the sample reported a previous pregnancy; although this is expected in this school-based adolescent population, it means that the items addressing prior pregnancy had no variability and could not contribute to the classification, which limits the applicability of that part of REND, an instrument originally developed for women aged 15 to 45 with or without a previous pregnancy. In the original version, items 11, 12 and 13 belong to the same factor. In the present analyses, items 11 and 12 remained together but loaded onto different factors, whereas item 13 loaded onto factor 1 in both analyses. Items 14 and 15 loaded onto factors 4 and 5 in FA, whereas in CATPCA they loaded onto factor 3. Item 16 also loaded together with items 14 and 15 on factor 3 in CATPCA. Based on the factor loadings obtained in each factor, one variable was selected from each retained factor:

From factor 1: REND i1; From factor 2: REND i2; From factor 3: REND i9; From factor 4: REND i16; From factor 5: REND i7.

REND i2 loaded above the suppression threshold in both analyses (0.77 in FA and 0.57 in CATPCA) but on different factors; it was retained as the marker of its dimension on conceptual grounds, since contraceptive use at first complete sexual intercourse is a central determinant of adolescent pregnancy risk, while we acknowledge that its factor placement was less stable than that of the other retained variables.

### 3.5. Logistic Classification Models

To this point in the study, there are three variables retained for IRENE and five variables for REND that contribute to the instrument-based risk classification. In accordance with the methodology section, the results of the logistic regression analyses conducted to analyze their classification performance regarding instrument-defined adolescent pregnancy risk are presented in Table 5 and Table 6.

In Table 5, B represents the regression coefficient and Sig. the significance for each category relative to its reference. According to the IRENE penalized logistic regression (Table 5), the adolescent’s living arrangement and the age at first complete sexual intercourse were the categories most strongly associated with the instrument-based risk classification. Relative to living with both parents, living with the mother (OR = 16.86, *p*-value = 0.002), with the father (OR = 106.95, *p*-value < 0.001), or with other relatives (OR = 35.95, *p*-value = 0.002) was associated with a higher probability of being classified at risk. Likewise, relative to adolescents who had not initiated complete sexual intercourse, every category of age at first intercourse was significantly associated with a higher risk classification (*p*-value < 0.05 in all cases). In contrast, the father’s educational level did not reach statistical significance in any category once the estimates were stabilized by penalization. Categories that did not reach statistical significance were not interpreted as increasing or reducing risk; only the statistically significant categories are discussed as associations.

The results of the REND binary logistic regression are presented in Table 6. Among the variables included, REND i1 showed a strong and statistically significant association with pregnancy risk classification (B = 3.47, *p*-value < 0.001). Given the inverse scoring of this variable, higher scores reflect earlier sexual initiation, indicating that earlier sexual debut is associated with a higher probability of belonging to the at-risk group.

REND i7, which reflects the adolescent’s response to a potential unplanned pregnancy, was also significantly associated with pregnancy-risk classification (B = 1.46, *p*-value = 0.039). For REND i9, perceiving the current situation as an appropriate time to become pregnant was associated with higher risk group classification, although this association was only marginally significant (B = 1.99, *p*-value= 0.050). In contrast, REND i2 and REND i16 were not significantly associated with pregnancy risk classification (*p*-value ≥ 0.050). As shown in Table 7, both logistic models showed high overall classification accuracy, with 96.9% for IRENE and 96.2% for REND. The Nagelkerke R2 values were 0.78 for IRENE and 0.86 for REND.

When the discrimination of the models was validated internally, the area under the ROC curve remained high and close to the apparent value, namely 0.95 (95% CI 0.90 to 0.99) for IRENE and 0.99 (95% CI 0.98 to 1.00) for REND under 10-fold cross-validation, which indicates limited optimism in discrimination. Importantly, these discrimination and accuracy values should be read as evidence of internal reproduction of the instrument-defined classification rather than as prospective predictive performance, because the predictors and the outcome derive from the same instruments. Nevertheless, the high overall accuracy and the Nagelkerke R^2^ partly reflect the marked class imbalance: for IRENE, with 41 at-risk cases, sensitivity was 81.0% against a specificity of 99.3%, so that the overall accuracy approached the proportion of adolescents classified as not at risk. The Firth penalized models, which retained all cases and avoided the unstable estimates caused by separation, confirmed the main associations, that is, earlier sexual initiation and the adolescent’s living arrangement for IRENE, and earlier sexual initiation together with the perceived appropriateness of the current moment for pregnancy for REND. Calibration was adequate for both models (Hosmer–Lemeshow *p* = 0.70 for IRENE and *p* = 0.07 for REND).

Beyond the cross-sectional analyses, a 12-month follow-up was attempted to describe pregnancy incidence: of the 319 adolescents, 127 (39.8%) were reached. Within this group, one adolescent reported a pregnancy, which ended in spontaneous abortion, and seven requested emergency contraception. Because the follow-up captured a single pregnancy event, it does not support outcome-based validation and is reported only descriptively, reinforcing the need for external validation with prospectively observed pregnancy outcomes.

## 4. Discussion

After cultural adaptation, both instruments showed favorable initial evidence of comprehensibility and content validity in Colombian adolescents. Adolescents reported high item comprehension in both IRENE and REND, although experts initially identified greater difficulties with IRENE. Content validity was also favorable for both instruments, suggesting that their items adequately represent the conceptual dimensions of the pregnancy risk construct. This level of expert agreement supports the relevance of the retained content and provides a solid foundation for this initial stage of validation [42].

Regarding instrument-defined pregnancy risk, the two instruments showed substantial overall agreement, particularly in the classification of adolescents without risk. At the same time, systematic differences were observed between them: IRENE tended to classify a greater proportion of adolescents in lower risk categories, whereas REND classified a larger proportion as at risk. Despite these differences, the high classification accuracy and the Nagelkerke R^2^ values suggest that both models are useful for reproducing the instrument-defined risk classification; their applied use as a screening tool, however, would require external validation against prospectively observed pregnancy outcomes.

From an operational perspective, these differences can be interpreted in light of the structure of the instruments, because in both cases the classification relies heavily on variables related to sexual behavior. In IRENE, four of the eight items address this domain, whereas in REND, this applies to eleven out of the fifteen analyzed items. Therefore, among adolescents who are not currently engaging in complete sexual intercourse, risk classification may be strongly influenced by the absence of behavioral indicators, which helps explain part of the observed concordance and discordance between the instruments. In addition, the factor structure obtained for REND departed substantially from its original version, and several items showed limited variability in this school-based sample; taken together, these findings may indicate that some of the original REND dimensions are not fully transferable to this adolescent Colombian population, which reinforces the need for future construct-validation studies in independent samples.

Across both instruments, age at first complete sexual intercourse emerged as the only shared contributor to pregnancy risk classification. This finding is consistent with the previous literature identifying early sexual debut as a key factor associated with adolescent pregnancy risk [43,44]. From a prevention perspective, this pattern supports the importance of delaying sexual debut and strengthening safe sex practices through sexuality education and access to contraceptives [45].

Analytically, one variable was retained from each dimension and then entered into the corresponding logistic model. In IRENE, the three variables retained were father’s educational level, current living arrangement, and age at first complete sexual intercourse; of these, the living arrangement and the age at first complete sexual intercourse showed the strongest associations with the instrument-based risk classification. In REND, the most relevant variables were age at first complete sexual intercourse, the decision the adolescent would make if pregnancy occurred, and the perception that the current moment was an appropriate time to become pregnant. It should be acknowledged, however, that retaining a single highest-loading indicator per dimension prioritizes statistical representativeness and may not preserve the full conceptual breadth of each latent construct; the retained items should therefore be regarded as parsimonious markers of their dimensions rather than as exhaustive representations of them, and their content coverage should be confirmed before applied use.

Beyond individual behavior, the findings also highlight the relevance of contextual and familial determinants of adolescent pregnancy risk. This is consistent with evidence showing that family structure and parent-adolescent communication influence sexual risk behavior and pregnancy outcomes [46]. Consistent with the IRENE model, living with both parents appears to exert a comparative protective effect, whereas single-parent arrangements are associated with an increased susceptibility to risk [47]. A plausible mechanism is that stronger parental monitoring and sexual communication can delay sexual initiation and support safer sexual decision-making [48]. Although the father’s educational level was retained as a structural marker, it was not independently associated with the risk classification once estimates were stabilized by penalization, which suggests that family structure, more than parental education alone, accounts for this contextual contribution.

The findings also reinforce the importance of sexual initiation and contraceptive context. Earlier and riskier sexual initiation has been consistently associated with adolescent pregnancy in the literature, and this aligns with the prominence of age at first complete sexual intercourse in both models. Although the REND variables referring to contraceptive use during first intercourse and source of contraceptive information were not statistically significant after adjustment, they remain conceptually relevant because limited contraceptive knowledge, poor-quality information, and barriers to access may increase vulnerability to unintended pregnancy [49,50].

Beyond individual knowledge, contraceptive use is also shaped by structural barriers, including cost, limited confidentiality, inadequate provider training, and the absence of adolescent-friendly services [50]. Under those conditions, adolescents may rely on peers or informal sources for contraceptive information, which may be incomplete or inaccurate. This may help explain why the source of contraceptive information emerged as a retained variable in REND, even if its adjusted association was not statistically significant.

Furthermore, the REND variables related to decision-making in the event of pregnancy and the perception that the current moment might be an appropriate time to become pregnant suggest that pregnancy intentions and attitudinal ambivalence remain relevant to adolescent risk classification. Prior research has shown that pregnancy intentions are often not fully dichotomous and that ambivalence can coexist with inconsistent contraceptive behavior and increased vulnerability to unintended pregnancy [51]. In that sense, these variables may capture not only reproductive intentions, but also broader expectations about support, timing, and future life conditions.

From an applied standpoint, a parsimonious set of items could facilitate the work of nurses, who attend adolescents across schools, primary care, community settings, and adolescent health services and are therefore well positioned to deliver brief risk screening and timely preventive care [52]. In these settings, a short instrument could support rapid triage within school health programs, opportunistic screening during routine primary care or adolescent visits, and the prioritization of adolescents who would benefit from counselling and referral to contraceptive services. Consistent with this view, structural interventions that strengthen the screening role of school nurses have been shown to increase adolescents’ receipt of sexual and reproductive health care [53]. Nevertheless, and in line with the cautions stated above, such applications would be appropriate only once the reduced models are validated against prospective outcomes in independent samples. This brevity involves a trade-off: reducing each multidimensional instrument to a few items prioritises rapid screening over the broader content coverage of the original tools, so the short form is best understood as a triage aid that flags adolescents for fuller assessment rather than as a replacement for the complete instruments.

It is necessary, finally, to acknowledge a methodological limitation that constrains the interpretation of these findings. Because the predictors and the outcome derive from the same instruments, the logistic models largely reproduce the scoring rules of IRENE and REND rather than predicting an independent outcome, a situation described in the methodological literature as circular analysis or double dipping [54]. For this reason, the high classification accuracy and the Nagelkerke R^2^ values reported here should be read as evidence of internal reproduction of the instrument classification, and not as evidence of predictive performance in real-world settings. Consistent with current guidance for prediction model studies, any claim of predictive validity would require internal validation through resampling and, above all, external validation in independent adolescent samples with prospectively observed pregnancy outcomes [55,56]. These steps were beyond the scope of the present study and constitute a priority for future research. Moreover, because participants were recruited from a single educational institution through non-probabilistic convenience sampling, the external validity of the findings is limited to comparable urban Colombian school populations, and the results should not be extrapolated to Latin American adolescents or to broader school populations without confirmation in probabilistic, multi-site samples. In addition, a 12-month follow-up was attempted to observe pregnancy outcomes, but the very low number of events recorded precluded any outcome-based validation.

### Limitations

Several limitations should be acknowledged. First, the analysis is circular, since the predictors and the outcome derive from the same instruments; consequently, the models reproduce the instrument scoring rather than predicting an independent outcome, and the high accuracy and the Nagelkerke R squared reflect internal reproduction rather than predictive performance. Second, the outcome is instrument-defined and was neither clinically verified nor prospectively observed, and no external validation was carried out, so external validation in independent samples with observed pregnancy outcomes is required before any applied use. Third, the cross-sectional design precludes causal inference, and therefore the associations reported for parental education, family structure, and sexual initiation should not be read as causal effects. Fourth, participants were recruited from a single educational institution through non-probabilistic convenience sampling, which restricts external validity to comparable urban Colombian school populations. Fifth, the small number of at-risk cases produced class imbalance and quasi-complete separation in some categories, yielding unstable coefficients that were mitigated with Firth penalized regression but should still be interpreted with caution. Sixth, the reliance on self-report for sensitive sexual and reproductive health questions may introduce social desirability and recall bias. Seventh, retaining a single item per dimension maximizes parsimony but may narrow content coverage. Finally, the REND factor structure departed from the original solution and the items addressing previous pregnancy showed no variability in this adolescent sample, which limits the construct coverage of that part of the instrument and warrants confirmation in broader, multi-site, and probabilistic samples. In addition, only face and content validity were assessed; construct, criterion, and test–retest validity, internal consistency, and measurement invariance were not examined, so these results represent an initial stage of cultural adaptation and validation rather than a full psychometric validation.

## 5. Conclusions

In conclusion, this study culturally adapted the IRENE and REND instruments for Colombian adolescents and, through dimensionality reduction, identified a minimal set of variables that reproduced the instrument-based pregnancy risk classification with high internal accuracy, with age at first complete sexual intercourse emerging as the only contributor shared by both instruments. Because the predictors and the outcome derive from the same instruments, these results should be read as evidence of internal reproduction of the instrument classification rather than as prediction of an observed pregnancy, and they require external validation in independent adolescent samples. With that caveat, a brief, culturally adapted set of items could support nurses in the rapid screening of adolescents at higher risk in school and primary care settings. The main limitations are the use of a single institution and non-probabilistic convenience sampling, which restrict the generalizability of the findings.

## Figures and Tables

**Table 1:** Sociodemographic characteristics of the adolescents (*n* = 319).

Demographic Variable	*n*	Percentage
Socioeconomic stratum		
Stratum 1	38	11.91
Stratum 2	80	25.08
Stratum 3	124	38.87
Stratum 4	76	23.82
Stratum 5	1	0.32
Religious belief		
Catholic	33	10.34
Non-Catholic Christian	207	64.89
None	59	18.50
Other	20	6.27
Grade		
Ninth	58	18.18
Tenth	256	80.25
Eleventh	5	1.57
Source of income (household)		
Mother	101	31.76
Father	50	15.72
Both	167	52.52
Sexual activity		
Has had sexual intercourse	64	20.1
Currently sexually active	30	9.4

Note. Socioeconomic stratum refers to the Colombian classification of residential dwellings into six strata (1 = lowest, 6 = highest), established for the differential charging of public utilities (Law 142 of 1994) and administered by the National Administrative Department of Statistics (DANE); lower strata correspond to more economically vulnerable households [29]. Religious belief, grade, and source of household income are self-reported categories.

**Table 2:** Cross-tabulation of the IRENE and REND risk classifications (counts and percentages).

	REND: Not at Risk	REND: At Risk	Total
IRENE: Not at risk	253 (80.3%)	21 (6.7%)	274 (87.0%)
IRENE: At risk	7 (2.2%)	34 (10.8%)	41 (13.0%)
Total	260 (82.5%)	55 (17.5%)	315 (100%)

Note. Cross-tabulation of the IRENE and REND risk classifications, based on the 315 adolescents with a valid classification on both instruments; counts and percentages are shown for each cell. In this two-by-two table, 41 adolescents were classified as at risk by IRENE; the IRENE regression model was fitted on the full IRENE sample, in which 41 adolescents were classified as at risk.

**Table 3:** IRENE: FA and CATPCA loading matrices.

Items Comprising IRENE	Factor Analysis			CATPCA		
	Factors			Factors		
	1	2	3	1	2	3
IRENE i6. With how many different people have you had complete sexual intercourse?	0.84			0.99		
IRENE i5. At what age was your first complete sexual intercourse? *	**0.91**			**0.99**		
IRENE i7. Do you use a condom for complete sexual intercourse?	−0.95			−0.86		
IRENE i1. What is your father’s level of education? *		**0.82**			**0.86**	
IRENE i2. What is your mother’s level of education?		0.76			0.81	
IRENE i8. Are you currently trying to get pregnant?						
IRENE i4. What are your family relationships like?			0.74			0.72
IRENE i3. Do you live with your parents? *			**−0.71**			**−0.78**
Explained variance (%)	25.1	17.8	14.1	36.3	19.4	15.5

Notes. FA = factor analysis; CATPCA = categorical principal component analysis. FA loadings are based on the rotated component matrix using Varimax rotation. Variables marked with bold letters showed high loadings in both FA and CATPCA and were selected for subsequent analyses. Blank cells indicate loadings below the suppression threshold of 0.40. Variables selected for the logistic models (one per dimension) are marked with an asterisk (*). IRENE item 8 loaded below 0.40 on all factors and was not retained. The values shown are factor and component loadings, that is, the correlation of each item with the corresponding factor (FA) or component (CATPCA), ranging from −1 to +1; higher absolute values indicate a stronger association of the item with the dimension [41].

**Table 4:** REND: FA and CATPCA loading matrices.

Items Comprising REND	FA					CATPCA				
	Factors					Factors				
	1	2	3	4	5	1	2	3	4	5
REND i1. At what age did you have your first complete sexual intercourse? *	**0.86**					**0.96**				
REND i2. Did you use any contraceptive method during your first complete sexual intercourse? *		**0.77**							**0.57**	
REND i3. Are you currently having complete sexual relations?	0.80					0.73				
REND i4. How often do you have complete sexual intercourse?	0.86					0.94				
REND i5. How many sexual partners have you had in the last six months?	0.82					0.91				
REND i6. Do you currently have a stable partner?	−0.70					−0.52				
REND i7. If you were to get pregnant without wishing to, what decision would you make? *					**0.63**					**0.76**
REND i8. In your current situation, would you like to get pregnant?			**0.80**				**0.62**			
REND i9. Do you think that, in your current situation, it would be the right time to get pregnant? *			**0.70**				**0.68**			
REND i11. How often do you use contraceptive methods for complete sexual intercourse?		0.76				0.64				
REND i12. Which contraceptive method do you use most often?		0.67				0.84				
REND i13. In your case, who chose the contraceptive method you use?	0.72					0.88				
REND i14. Whether you use contraception or not, which contraceptive method do you consider the safest to prevent pregnancy?				0.58				0.51		
REND i15. Please rate the usefulness of information you have received about sexuality and contraception.					0.63			0.55		
REND i16. Where did you get the information, you have concerning contraception? *				**0.80**				**0.58**		
**Explained variance (%)**	**28.5**	**14.7**	**8.4**	**8.1**	**7.3**	**37.4**	**8.9**	**8.3**	**7.5**	**6.9**

*Notes:* FA = Factor Analysis; CATPCA = Categorical Principal Component Analysis. FA values are based on the rotated component matrix using Varimax rotation. Variables marked with bold letters had high loadings in both FA and CATPCA and were selected for subsequent analyses. Blank cells indicate loadings below the suppression threshold of 0.40. Variables selected for the logistic models (one per dimension) are marked with an asterisk (*). The values shown are factor and component loadings, that is, the correlation of each item with the corresponding factor (FA) or component (CATPCA), ranging from −1 to +1; higher absolute values indicate a stronger association of the item with the dimension [41].

**Table 5:** Binary logistic regression model outcomes for IRENE: predicted group membership.

Variables	B	SE	OR	95% CI	Sig.
Father’s level of education (IRENE i1) a					
Does not know	1.02	1.12	2.77	0.31, 24.95	0.364
Primary/Elementary	0.11	0.91	1.11	0.19, 6.63	0.907
Secondary	1.39	1.10	4.03	0.46, 34.96	0.206
High school	0.27	0.84	1.31	0.25, 6.73	0.748
Technical studies	1.19	1.09	3.28	0.39, 27.97	0.277
Do you live with your parents? (IRENE i3) b					
Mother	2.83	0.90	16.86	2.90, 97.93	0.002
Father	4.67	1.25	106.95	9.26, 1235.19	<0.001
With family	3.58	1.17	35.95	3.65, 353.84	0.002
At what age was your first complete sexual intercourse? (IRENE i5) c					
Under 14 years old	4.36	1.17	78.49	7.87, 782.49	<0.001
At the age of 14	6.38	1.10	590.38	68.06, 5121.22	<0.001
At the age of 15	6.36	0.99	576.80	82.93, 4011.88	<0.001
At the age of 16	2.62	0.94	13.76	2.18, 86.79	0.005
At age 17 or older	4.52	2.23	91.97	1.15, 7342.42	0.043
Constant	−7.03	1.08	0.00	0.00, 0.01	<0.001

Notes. a Reference category = university. b Reference category = living with both parents. c Reference category = I have not had complete sexual intercourse. B = regression coefficient; SE = standard error; OR = odds ratio; CI = confidence interval; Sig. = significance based on the Wald statistic. Estimates were obtained by Firth penalized maximum likelihood to address the quasi-complete separation produced by the small number of at-risk cases; odds ratios are reported with Wald-type 95% confidence intervals.

**Table 6:** Binary logistic regression model outcomes for REND: predicted group membership.

Variables	B	SE	OR	95% CI	Sig.
Age at first complete sexual intercourse (REND i1)	3.47	0.47	32.22	12.73, 81.55	<0.001
Contraceptive use at first complete sexual intercourse (REND i2)	1.64	1.58	5.15	0.23, 113.33	0.299
Decision facing an unintended pregnancy (REND i7)	1.46	0.71	4.32	1.08, 17.36	0.039
Perceived right time to become pregnant (REND i9)	1.99	1.02	7.34	1.00, 53.86	0.050
Source of contraceptive information (REND i16)	1.83	1.04	6.23	0.81, 47.71	0.078
Constant	−11.48	2.48	0.00	0.00, 0.00	<0.001

Notes. B = regression coefficients; Sig. = significance based on Wald statistics. Variables correspond to the five REND items selected from the structural analyses. Each REND item was entered as a single ordinal predictor, that is, one coefficient per item reflecting its original scoring, whereas the IRENE items were entered as categorical predictors with reference categories. Estimates were obtained by Firth penalized maximum likelihood, and odds ratios are reported with Wald-type 95% confidence intervals.

**Table 7:** Classification accuracy (Confusion Matrices) for the models.

Observed Group	Predicted by IRENE			Predicted by REND		
	Groups IRENE		Correct Percentage	Groups REND		Correct Percentage
	Without Risk	with Risk		Without Risk	with Risk	
Without risk	274	2	99.3	251	9	96.5
With risk	8	34	81.0	3	52	94.5
Overall percentage			96.9			96.2

Note. Nagelkerke R^2^ = 0.78 for IRENE and 0.86 for REND.

## Data Availability

The data presented in this study are available on request from the corresponding authors.

## Supplementary Materials

The following supporting information can be downloaded at: https://www.mdpi.com/article/10.3390/nursrep16070248/s1, Table S1: Adjustments made to the IRENE and REND items (original vs Colombian version); Table S2: Item-level content validity index (CVI) of IRENE and REND; Table S3: IRENE: items, dimensions and risk-classification rule; Table S4: REND: items, scoring direction and risk threshold.

## Public Involvement Statement

No members of the public or patients were involved in the design, conduct, analysis, reporting, or dissemination plans of this research.

## Guidelines and Standards Statement

This manuscript was prepared in accordance with the Strengthening the Reporting of Observational Studies in Epidemiology (STROBE) guidelines for cross-sectional research, incorporating established methodological standards for cultural adaptation, as well as face and content validation.
