# Supplementary material for: Cultural Adaptation and Selection of a Minimal Set of Variables from Two Adolescent Pregnancy Risk Instruments (IRENE and REND) in Colombian Schoolgirls
Source: Nurs Rep. 2026 Jul 16;16(7):248. doi: 10.3390/nursrep16070248 (PMC13414866; doi:10.3390/nursrep16070248)
Supplement: Supplementary file 1 [file nursrep-16-00248-s001.zip › nursrep-4365929-supplementary.pdf]

Supplementary Materials

Cultural adaptation of IRENE and REND (Colombian version)

Table S1. Adjustments made to the IRENE and REND items (original vs Colombian version)

**Instrument IRENE**	
**Original version**	**Colombian version**
Item 4. ¿Cómo estás de satisfecha con tus relaciones familiares?	¿Cómo son sus relaciones familiares?
Item 5. ¿Qué edad tenías cuando tuviste tu primera relación sexual completa?	¿A qué edad fue su primera relación sexual completa?
Item 7. ¿Utilizáis tú y tu pareja preservativo?	¿Utilizas preservativo en tus relaciones sexuales completas?
**Instrument REND**	
**Original version**	**Colombian version**
Items 1, 2, 3, 4, 11 and the clarifying phrases mentioning “coital”.	Changed to “completa” (complete).
Item 1, clarifying phrase: “varón”; the phrase “eyaculación vaginal, con o sin orgasmo” was removed.	“varón” changed to “hombre”.
Item 2, clarifying phrase “Entendiéndose como método anticonceptivo, cualquier acto, dispositivo o medicación para impedir un embarazo”.	Modified to “Entendiéndose por método anticonceptivo, cualquier tipo de anticoncepción utilizado para evitar un embarazo”.
Item 4. Habitualmente, ¿con qué frecuencia mantiene relaciones sexuales coitales?	Habitualmente, o en los últimos 6 meses…
Items 11, 12 and 13 (response options).	Added the response option: “No he tenido nunca relaciones sexuales completas”.
Items 13 and 16, response options referring to a health professional/centre: “sanitario”.	Changed to “salud”.
Items 11, 12 and 14, response options: “marcha atrás”, “DIU Cobre/Hormonal”, “implante”.	Changed/added to “coito interrumpido”, “Dispositivo intrauterino”, “implante subdérmico (barras)”.

Source: authors’ database; adaptation by the authors.

Table S2. Item-level content validity index (CVI) of IRENE and REND

Note. CVI computed from the expert panel ratings. I-CVI = item-level content validity index; S-CVI/Ave = scale-level CVI averaged across items. No item fell below the exclusion threshold; all items were retained.

IRENE (8 items)

Item	I-CVI Pertinence	I-CVI Relevance
Father's educational level	1.00	1.00
Mother's educational level	1.00	1.00
Living arrangement (living with parents)	0.94	0.94
Family relationships	1.00	1.00
Age at first complete sexual intercourse	0.94	1.00
Number of sexual partners	0.94	0.94
Condom use	1.00	1.00
Seeking pregnancy at present	1.00	1.00
**S-CVI/Ave**	**0.98**	**0.99**

REND (16 items)

Item	I-CVI Pertinence	I-CVI Relevance
Age at first complete sexual intercourse	0.85	0.85
Contraceptive use at first intercourse	0.85	0.85
Currently has complete intercourse	0.85	0.85
Frequency of intercourse	0.80	0.80
Sexual partners in the last 6 months	0.80	0.85
Stable partner	0.85	0.85
Decision facing an unintended pregnancy	0.80	0.85
Wish to become pregnant	0.85	0.85
Perceived right time to become pregnant	0.85	0.85
Whether a previous pregnancy was planned	0.85	0.85
Frequency of contraceptive use	0.80	0.85
Most frequently used contraceptive method	0.80	0.85
Who chose the contraceptive method	0.75	0.80
Method perceived as safest	0.80	0.80
Usefulness of the information received	0.75	0.70
Source of contraceptive information	0.85	0.85
**S-CVI/Ave**	**0.82**	**0.83**

Scoring of the culturally adapted IRENE and REND instruments

Note. Item wording follows the culturally adapted instruments. The response options and their numeric coding for IRENE are listed in the table; the non-applicable option for adolescents who had never had complete sexual intercourse was scored as zero. For IRENE, risk is determined at the dimension level according to the classification rule described in the Methods; for REND, the item scores are summed, with a cut-off of 12.

Table S3. IRENE: items, dimensions and risk-classification rule

Item	Dimension	Response options / coding	Direction toward risk
i1. What is your father's level of education?	1. Parents' educational level	0 = Don't know / No answer; 1 = Primary; 2 = Secondary; 3 = High school; 4 = Technical; 5 = University	Lower education increases risk
i2. What is your mother's level of education?	1. Parents' educational level	0 = Never studied / illiterate; 1 = Primary; 2 = Secondary; 3 = High school; 4 = Technical; 5 = University; 6 = Don't know / No answer	Lower education increases risk
i3. Do you live with your parents?	2. Family APGAR	1 = With father and mother; 2 = With mother; 3 = With father; 4 = With other relatives; 5 = None of the above	Not living with both parents increases risk
i4. What are your family relationships like?	2. Family APGAR	0 = Don't know / No answer; 1 = Bad; 2 = Acceptable; 3 = Good; 4 = Very good	Poorer family relationships increase risk
i5. At what age was your first complete sexual intercourse?	3. Sexual behavior	0 = Never had complete sexual intercourse; 1 = Under 14 years; 2 = 14 years; 3 = 15 years; 4 = 16 years; 5 = 17 years or older	Earlier age increases risk
i6. With how many different people have you had complete sexual intercourse?	3. Sexual behavior	Numeric count of partners reported by the participant	More partners increase risk
i7. Do you use a condom for complete sexual intercourse?	4. Contraceptive use	0 = Do not use it because I do not have complete intercourse; 1 = Yes, I always use a condom; 2 = I usually use a condom, although sometimes we forget; 3 = I usually use a condom, sometimes forget, but use other methods; 4 = I rarely use a condom, but use other methods; 5 = I rarely use a condom and do not use other methods; 6 = I do not use it	Non-use increases risk
i8. Are you currently trying to get pregnant?	5. Pregnancy desire	0 = Don't know / No answer; 1 = Strongly disagree; 2 = Disagree; 3 = Agree; 4 = Strongly agree	Desire/intention increases risk

Risk classification (G2 = at risk). An adolescent is classified as at risk when at least three of the five dimensions are affected; when two affected dimensions include sexual behavior, contraceptive use or pregnancy desire; or when the affected dimensions correspond to the combinations (3 and 4), (3 and 5), (4 and 5), (1, 2 and 3), (1, 2 and 4) or (1, 2 and 5).

Table S4. REND: items, scoring direction and risk threshold

Item	Dimension	Direction toward risk
i1. At what age did you have your first complete sexual intercourse?	Sexual behavior and habits	Earlier age increases risk
i2. Did you use any contraceptive method during your first complete sexual intercourse?	Contraceptive knowledge and use	Non-use increases risk
i3. Are you currently having complete sexual relations?	Sexual behavior and habits	Yes increases risk
i4. How often do you have complete sexual intercourse?	Sexual behavior and habits	Higher frequency increases risk
i5. How many sexual partners have you had in the last six months?	Sexual behavior and habits	More partners increase risk
i6. Do you currently have a stable partner?	Sexual behavior and habits	Coding per instrument
i7. If you were to get pregnant without wishing to, what decision would you make?	Pregnancy intention	Per instrument coding
i8. In your current situation, would you like to get pregnant?	Pregnancy intention	Desire increases risk
i9. Do you think that, in your current situation, it would be the right time to become pregnant?	Pregnancy intention	Perceived appropriateness increases risk
i10. If you have been pregnant before, was the pregnancy planned?	Pregnancy intention	Excluded (no variability in the sample)
i11. How often do you use contraceptive methods for complete sexual intercourse?	Contraceptive knowledge and use	Less frequent use increases risk
i12. Which contraceptive method do you use most often?	Contraceptive knowledge and use	Per instrument coding
i13. In your case, who chose the contraceptive method you use?	Contraceptive knowledge and use	Per instrument coding
i14. Which method do you consider safest to prevent pregnancy?	Contraceptive knowledge and use	Per instrument coding
i15. Rate the usefulness of information received about sexuality and contraception.	Contraceptive knowledge and use	Per instrument coding
i16. Where did you get the information you have concerning contraception?	Contraceptive knowledge and use	Per instrument coding

Scoring. The REND total score is the sum of the 16 item scores, with higher scores indicating greater risk. An adolescent is classified as at risk (G2) when the total score is greater than or equal to 12 (confirmed by the first author).
